# Challenges in, and recommendations for, hyperspectral imaging in ex vivo malignant glioma biopsy measurements

**DOI:** 10.1038/s41598-023-30680-2

**Published:** 2023-03-07

**Authors:** Anna Walke, David Black, Pablo A. Valdes, Walter Stummer, Simone König, Eric Suero-Molina

**Affiliations:** 1 Department of Neurosurgery, University Hospital of Münster, Albert-Schweitzer-Campus 1, A1, 48149 Münster, Germany; 2 Core Unit Proteomics, Interdisciplinary Centre for Clinical Research, University of Münster, Münster, Germany; 3 Department of Electrical and Computer Engineering, University of British Columbia, Vancouver, Canada; 4 Department of Neurosurgery, University of Texas Medical Branch, Galveston, TX USA

**Keywords:** Biological fluorescence, Medical imaging, Brain imaging

## Abstract

The visualization of protoporphyrin IX (PPIX) fluorescence with the help of surgical microscopes during 5-aminolevulinic acid-mediated fluorescence-guided resection (FGR) of gliomas is still limited at the tumor margins. Hyperspectral imaging (HI) detects PPIX more sensitively but is not yet ready for intraoperative use. We illustrate the current status with three experiments and summarize our own experience using HI: (1) assessment of HI analysis algorithm using pig brain tissue, (2) a partially retrospective evaluation of our experience from HI projects, and (3) device comparison of surgical microscopy and HI. In (1), we address the problem that current algorithms for evaluating HI data are based on calibration with liquid phantoms, which have limitations. Their pH is low compared to glioma tissue; they provide only one PPIX photo state and only PPIX as fluorophore. Testing the HI algorithm with brain homogenates, we found proper correction for optical properties but not pH. Considerably more PPIX was measured at pH 9 than at pH 5. In (2), we indicate pitfalls and guide HI application. In (3), we found HI superior to the microscope for biopsy diagnosis (AUC = 0.845 ± 0.024 (cut-off 0.75 µg PPIX/ml) vs. 0.710 ± 0.035). HI thus offers potential for improved FGR.

## Introduction

Fluorescence-guided resection (FGR) with 5-aminolevulinic acid (5-ALA) is routinely used during malignant glioma surgery^1^. To that end, 5-ALA is administered orally to patients before surgery, which leads to the selective accumulation of protoporphyrin IX (PPIX) in glioma tissue^2^. The surgeon can then visualize PPIX in tumors by their red-pink fluorescence using appropriate, commercially available microscopes equipped with filter systems. Using FGR, the rate for complete resection of contrast-enhancing tumor was twice as high as for surgery under white light (65% vs. 36%)^1^. Together with ultrasound, neuronavigation, as well as mapping and monitoring techniques, a gross total resection can be achieved^3,4^.

The mechanisms that lead to 5-ALA-induced PPIX accumulation in cancer cells are multifactorial and not entirely clear^2,5^. Cellular uptake of 5-ALA is an important factor^6–8^. In that respect, passive diffusion or, more importantly, protein-mediated specific active transport are considered^5^. Moreover, blood–brain barrier (BBB) permeability is crucial, as an intact BBB is impermeable to 5-ALA^2^. Synthesis of PPIX after exogenous 5-ALA administration is thus predominantly observed in brain regions without BBB, such as the choroid plexus, or in case of disrupted BBB, as in high-grade glioma (HGG)^2,9,10^. Once 5-ALA enters the cell, alterations in heme biosynthesis and features of the tumor microenvironment^5^, like altered glucose metabolism and hypoxia, may trigger increased PPIX accumulation in cancer compared to healthy cells. Reduced expression and activity of the enzyme ferrochelatase (FECH) and iron deficiency are commonly observed in cancer^2,5,11^. FECH catalyzes the final step of heme biosynthesis, the insertion of a ferrous-(II)-ion into the core of PPIX. Both reduced FECH expression/activity and iron deficiency would result in PPIX accumulation, but scientific evidence is missing that this plays a role in 5-ALA-induced PPIX accumulation in vivo^2,5^.

Regardless of the mechanisms for selective PPIX formation, the tumor can be identified by its vibrant fluorescence using a commercial surgical microscope equipped with a dedicated filter system^12^. Such microscopes allow a qualitative description of the fluorescence emissions. The surgeon typically grades the fluorescence into “none”, “weak” or “strong” based on the observed intensity of the red-pink color compared to the violet-blue background. For more accurate measurements of the fluorescence intensity in tissues, spectrally resolving technologies^13–28^ are needed. They serve as a research tool, e.g., to perform PPIX quantification in tissues with no visible fluorescence, to optimize the timing of 5-ALA administration before surgery, to explore the potential of FGR for low-grade glioma surgery, and as an orthogonal method outside the operating room to test and validate new devices under development for FGR^18–20,29,30^. Currently, the technology is not widely employed, with only a few groups researching in a neurosurgical context^25,27,31–33^.

These spectroscopic devices are non-commercial, either single-point probe devices^15,17,25–27,32,34,35^ or wide-field hyperspectral imaging (HI) systems^16,18–24,28^. The former detects the spectrally resolved light from one point in the field of view (e.g., 1 mm diameter) using a probe in contact with tissue. The output is a single spectrum per point of detection^32,34^. The latter, in contrast, measures the spectrally resolved light from each point throughout the field of view (e.g., 1 cm diameter) without tissue contact. The output is a three-dimensional (3D) image, where each pixel contains spectral data, e.g., a fluorescence spectrum. Both instrumentation and software for signal processing are still under investigation, aiming at more sensitive and specific fluorescence detection and quantification of fluorophores in tissue, ultimately benefiting the patient^16^. Here, we discuss wide-field imaging-based hyperspectral detection systems^13–28^.

Hyperspectral measurements of PPIX in tissue allow correction for the confounding, non-linear effects of tissue optical properties at the excitation and emission wavelengths (i.e., absorption and scattering). Depending on the degree of such effects, two tissue regions with the same concentration of PPIX can present with significantly different raw fluorescence intensities, leading to inaccurate PPIX estimates^16,28,35–37^. Hyperspectral detection of reflectance at multiple wavelengths (e.g., excitation and emission) and fluorescence emissions can be used as input for correction algorithms to account for the effects of tissue optical properties on the detected signal^14,16,18,24,28,36,38^. In clinical practice, tumor tissue is revealed by this method that would otherwise have gone undetected, thus decreasing the number of false negative tumor identifications^25–27,39^.

Tissue autofluorescence also influences the detected fluorescence signal, leading to over- or underestimation of the PPIX contribution. Unless corrected, it can cause false-positive conclusions in the operating theatre and follow-up hyperspectral measurements^24^. Autofluorescence originates from compounds intrinsic to tissue such as collagen, tryptophan, lipofuscin, flavins, lipo-pigments, and free and protein-attached nicotinamide adenine dinucleotide (NADH)^36,40^. HI provides sufficient spectral resolution to enable the mathematical separation of the individual spectral contributions from autofluorescence, PPIX, and PPIX photo products^24,28^.

**Figure 1:**
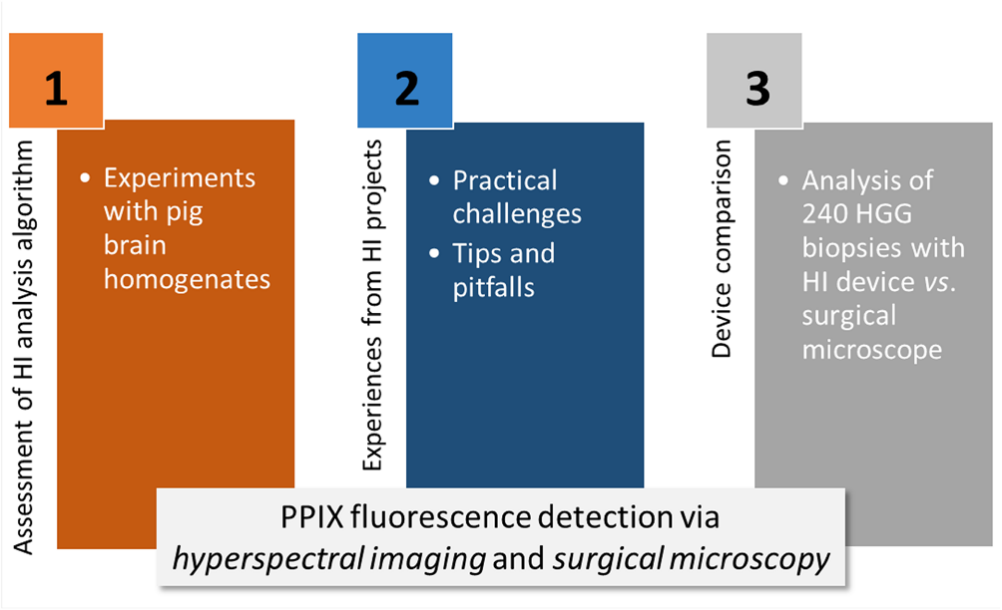
Fluorescence measurements in glioma by HI and surgical microscopy. Outline of this article, which evaluates the current status of both techniques, particularly of HI, the more sensitive of the two.

The visualization of PPIX fluorescence by surgical microscopy during FGR of glioma is highly limited at the tumor margins, respectively, at the infiltration zone. HI is capable of more specific and sensitive PPIX detection. The technique has a high potential for future use during surgery but is still under development. This article discusses its advantages, limitations, and possible improvements for glioma biopsy diagnosis. The content of this article is based on different experiments and the authors' own experience (Fig. 1): (1) assessment of the reliability of current HI analysis algorithm^28,38,41^ using PPIX-spiked pig brain tissue, (2) evaluation of the practical challenges during ex vivo HI based on our experience from former projects^18–24,30^ and (3) analysis of the diagnostic accuracy for comparison of a commercially available surgical microscope and a wide-field HI system^16,18–24,28^ based on data from 240 HGG biopsies.

## Methods

### Surgical microscopy

The commercially available surgical microscope KINEVO 900 equipped with the BLUE 400 filter system (Carl Zeiss Meditec (CZM) AG, Oberkochen, Germany) was used in the operating theatre to visualize PPIX fluorescence in vivo.

### Hyperspectral imaging

**Figure 2:**
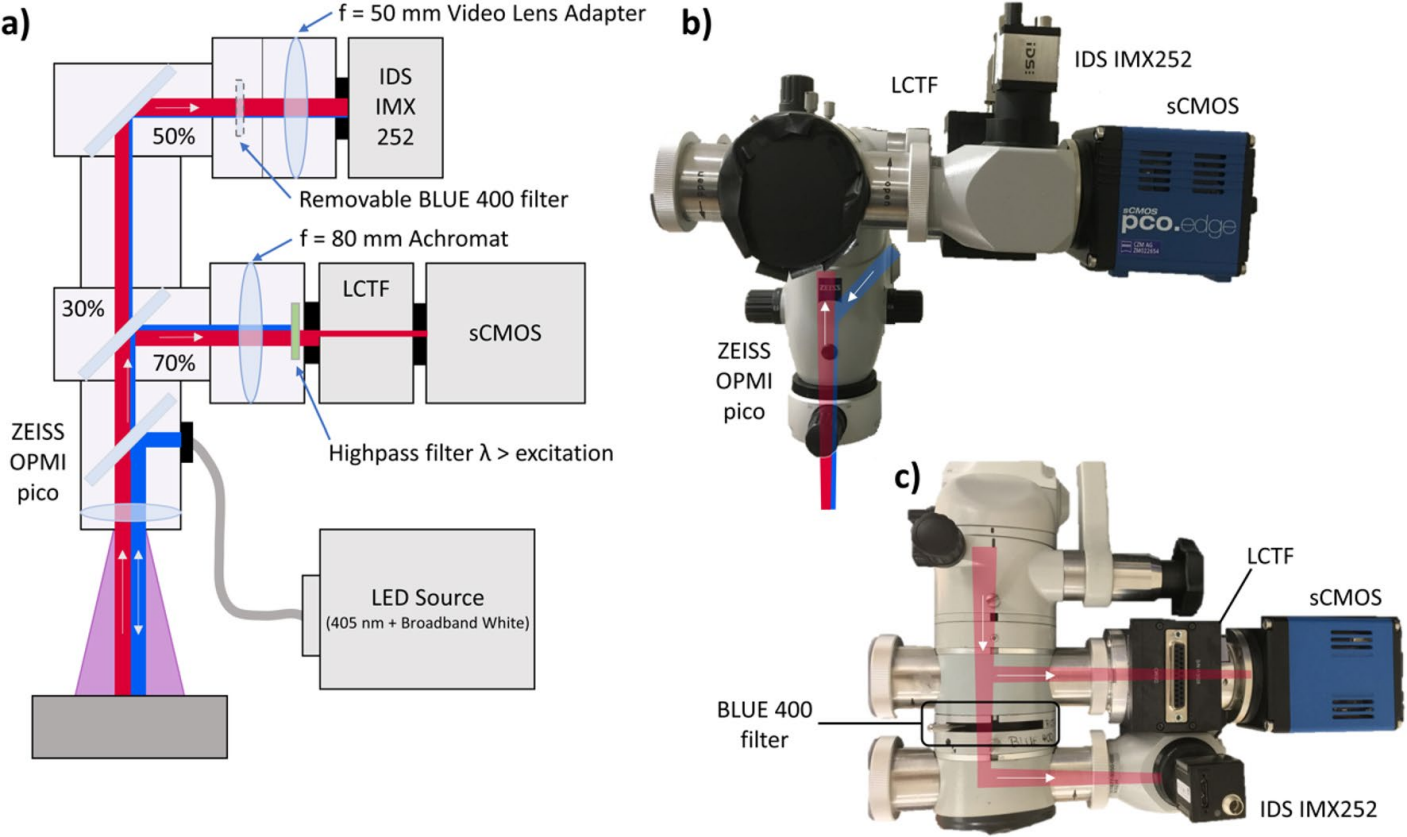
(**a**) Schematic illustration, (**b**) front view, (**c**) top view of the hyperspectral system used for ex vivo fluorescence imaging. Excitation light at 405 nm and broadband white light were delivered by an LED source (blue, bold). The emitted light (red, bold) from the sample was detected using the sCMOS camera after passing the LCTF, while the reflected light (blue, narrow) was filtered out by a highpass filter. An IDS color camera was additionally used to create a color image if desired with the BLUE 400 filter.

The technical setup is visualized in Fig. 2. The hyperspectral camera was built using an OPMI pico microscope (CZM, Oberkochen, Germany, Fig. 2). The system uses a 405 nm light-emitting diode (LED) for excitation, a scientific complementary metal–oxide–semiconductor (sCMOS) camera (PCO edge 4.2, PCO, Kehlheim, Germany), a liquid crystal tunable filter (LCTF, Meadowlark Optics Inc., Colorado, USA) and a color camera (IDS Imaging Development Systems GmbH, Obersulm, Germany) with an IMX252 sensor (Sony, Tokyo, Japan). Light was detected wavelength-dependent by placing the LCTF between an achromatic lens and the sCMOS camera. Residual light was additionally recorded with the IDS camera, resulting in a color image. A filter wheel with a BLUE 400 filter (CZM, Oberkochen, Germany) was installed before the color camera to create a BLUE 400 image decoupled from the hyperspectral measurements, which enabled reconstruction of a qualitative fluorescence image as observed in the conventional surgical microscope.

All camera components were controlled using custom LabView software (National Instruments Inc., Austin, Texas). For each biopsy, a hyperspectral 3D-image cube was acquired of the fluorescence emission (excitation wavelength 405 nm) from 421 to 730 nm with an increment of 3 nm. Similarly, a white light (excitation wavelengths 400–750 nm) hyperspectral 3D-image cube was measured. The white light reflectance spectrum was collected from 420 to 730 nm for each pixel with an increment of 5 nm. Then, a background image cube was acquired from 421 to 730 nm with an increment of 3 nm with the light source turned off to remove dark noise from the white and fluorescence cubes. Exposure times for acquisition of all three image cubes were fixed at 100 ms per wavelength, resulting in approximately three minutes for data acquisition in one sample. A 10 × 10-pixel binning and averaging were performed before analysis of the spectra. The effective area size of one pixel in the field of view was estimated experimentally, using a sheet of squared paper, by taking the difference of the spatial x/y-coordinates. The area of one 10 × 10 binned pixel in the digital image was about 210 × 210 µm (0.0441 mm^2^). Measurements were standardized daily by acquiring a white light reflectance test using a diffuse reflectance target (spectralon®, Labsphere, North Sutton, United States) with the same settings as for biopsy measurement, except for the exposure time at each wavelength, which was set to 50 ms. Subsequently, spectra of a non-bleaching fluorescence reference plate (fluorescence target BLUE 400, CZM, Oberkochen, Germany) were acquired with the same settings as for biopsy measurement, except for the exposure time at each wavelength, which was set to 40 ms for fluorescence and dark spectra, and 50 ms for the white light spectrum. Custom MATLAB (The Math Works Inc., Natick, Massachusetts) software was used to correct tissue optical properties with the algorithm previously developed by Valdes et al.^28,38,41^. This empiric algorithm uses the reflectance at the excitation and emission wavelengths to correct the fluorescence emissions for the distorting effects of tissue optical properties to calculate an absolute fluorophore contribution (for illustration of analysis steps, see supplementary Figure S1).

The hyperspectral imaging system was calibrated using tissue-mimicking liquid phantoms of known PPIX concentrations (0.0, 0.2, 0.6, 2.5 µg/ml) and varying optical properties (absorption at 405 nm: µ_a,405 nm_ = 18, 42, 60 cm^-1^; reduced scattering at 635 nm: µ’_s,635 nm_ = 8.7, 11.6, 14.5 cm^-1^), as described by Valdes et al.^26,28,35^. As in a prior work^24^, we refer to PPIX contributions rather than concentrations and report these in units of micrograms per milliliter to appreciate the inter-device variability between different groups^18,19,26,42^ and the calibration with phantoms^26,28,35^, which may not be adequate to determine absolute PPIX tissue concentrations due to nonlinearities in PPIX fluorescence^24,31^ (see below for validation with tissue homogenates).

The entire biopsy was chosen for analysis using the selection tool of the software. First, the PPIX contribution was calculated at every pixel in that area. Then these values were averaged to generate a single PPIX value for the whole biopsy. Only pixels with contributions greater than 0.1 µg/ml were included in the average calculation. This threshold was derived from experiments with pig brain homogenates, where the maximum of the native PPIX contribution was detected at this level. This threshold turned out to be a suitable measure to distinguish PPIX from the native background, which is essential for analyzing biopsies with a heterogeneous PPIX distribution.

### Preparation of pig brain homogenates

Experiments with pig brains were permitted by the Health and Veterinary Office Münster (Reg.-No. 05 515 1052 21). Pig brain was obtained from a local butcher and separated into the following anatomical parcels: cerebrum, cerebellum, hypothalamus, and brain stem/spinal cord. Each tissue section was washed with distilled water, roughly cut into 10 × 10 × 10 mm pieces and homogenized using a blender (VDI 12, VWR International, Hannover, Germany). Homogenates were stored at − 20 °C. For adjustment of pH to 5–9, 0.5 M tris(hydroxymethyl)aminomethane (Tris-base, Serva, Heidelberg, Germany) buffer was prepared with hydrochloric acid (HCl, Honeywell Riedel–de Haen, Seelze, Germany). Reference tissue homogenates (RTHs) with controlled pH (pH-RTHs) were composed of RTHs and buffer (w/v), as displayed in supplementary Table S1 before spiking of PPIX. For the preparation of RTHs without pH control (pH ~ 7), 200 to 600 mg of the homogenates were directly spiked with PPIX (Enzo Life Sciences GmbH, Lörrach, Germany) stock solution (300 pmol/µl in dimethyl sulfoxide, Merck KGaA, Darmstadt, Germany) to the desired concentrations (0.0, 0.5, 0.75, 1.0, 2.0, 3.0 and 4.0 pmol/mg) and homogenized using a vortex mixer. RTHs and pH-RTHs were transferred to a Petri dish forming tissue samples of about 4 × 4 × 2 mm. Hyperspectral measurements were performed immediately using the same parameters as for tissue biopsies. The software calculated the PPIX contribution in µg/ml based on the calibration with liquid phantoms^18–20,25–28^. A unit related to the sample weight is superior and more common in solid tissue samples like homogenates or brain biopsies, because these samples are routinely weighed for analysis in the laboratory. Thus, we refer to pmol/mg for the spiked samples of homogenates experiments and evaluate them in relation to the calculated PPIX contribution from HI in µg/ml. All generated raw data analyzed during this study are included in the supplementary data file.

### Patients

We analyzed 240 biopsies obtained from a cohort of 26 patients undergoing surgery for lesions suspicious of HGG (Table 1) using two different imaging devices: the surgical microscope KINEVO 900 and a modified OPMI pico microscope (Fig. 2) for HI. We here evaluate the hyperspectral data using a sample cohort, of which primary data was published in part earlier^30^.

**Table 1:** Overview of the patient cohort^30^. Patients and biopsies were classified according to 2016 World Health Organization (WHO) criteria^43^ (isocitrate dehydrogenase (IDH) mutant or wild type, O^6^-methylguanine-DNA-methyltransferase (MGMT) positive/methylated or negative/not-methylated) and according to the standardized performance score by the Eastern Cooperative Oncology Group (ECOG)^44^.

		Patients	%	Biopsies	%
Number		26		240	
Gender	Male	17	65	159	66
	Female	9	35	81	34
Age	Mean (SD), Range	60.8 ± 9.5, 37—75			
Histology	Anaplastic astrocytoma IDH-mutant, MGMT positive	1	4	10	4
	Glioblastoma IDH-wild type, MGMT positive	13	50	124	52
	Glioblastoma IDH-wild type, MGMT negative	10	38	86	36
	Glioblastoma IDH-mutant, MGMT positive	2	8	20	8
ECOG score	1	16	62	144	60
	2	10	38	96	40
Primary tumor		16	62	150	63
Recurrent tumor		10	38	90	37

A standard dose of 20 mg/kg of 5-ALA (Gliolan®, medac, Wedel, Germany) was orally administered four hours before induction of anesthesia. Biopsies were collected from the non-contrast enhancing, FLAIR positive, infiltrative tumor margins during surgery. Nine biopsies were taken on average per patient (average: 9.2 ± 1.5; range: 5–10 biopsies per patient). The fluorescence quality in the surgical microscope was rated by an experienced neurosurgeon in the categories “none”, “weak”, and “strong” as described previously^17^.

Methods were carried out in accordance with relevant guidelines and regulations. All experiments were approved by the local ethics committee of the University of Münster (2020-644-f-S) and informed consent was obtained from all patients.

### Neuropathology

Samples were analyzed at the Institute of Neuropathology, University Hospital of Münster, by a neuropathologist blinded to the intraoperative fluorescence data. Patients were operated on in 2020. Thus, 2016 WHO grading system was implemented for classification, and tissues were stained with hematoxylin and eosin (H&E) and elastic van Gieson. Furthermore, immunohistochemical and molecular analysis of IDH mutations, glial fibrillary acidic protein (GFAP), MGMT methylation, and Ki-67/MIB1 proliferation index were included. Biopsies were classified as reactively altered brain tissue (RABT), infiltration zone (IZ), or solid tumor (ST).

### Statistical analysis

Statistics were calculated using SPSS software (Version 27, IBM, Germany). Data distribution was evaluated using histograms and Kolmogorov–Smirnov test. As variables were not normally distributed, a Mann–Whitney U test (MWU) was used to compare groups. All reported *p*-values were two-tailed. A *p*-value < 0.05 was considered statistically significant. For pairwise comparison of receiver operating characteristic curves, MedCalc® Statistical software (version 20.111, MedCalc Software, Ostend, Belgium) was used.

## Results

### Assessment of HI analysis algorithm

**Figure 3:**
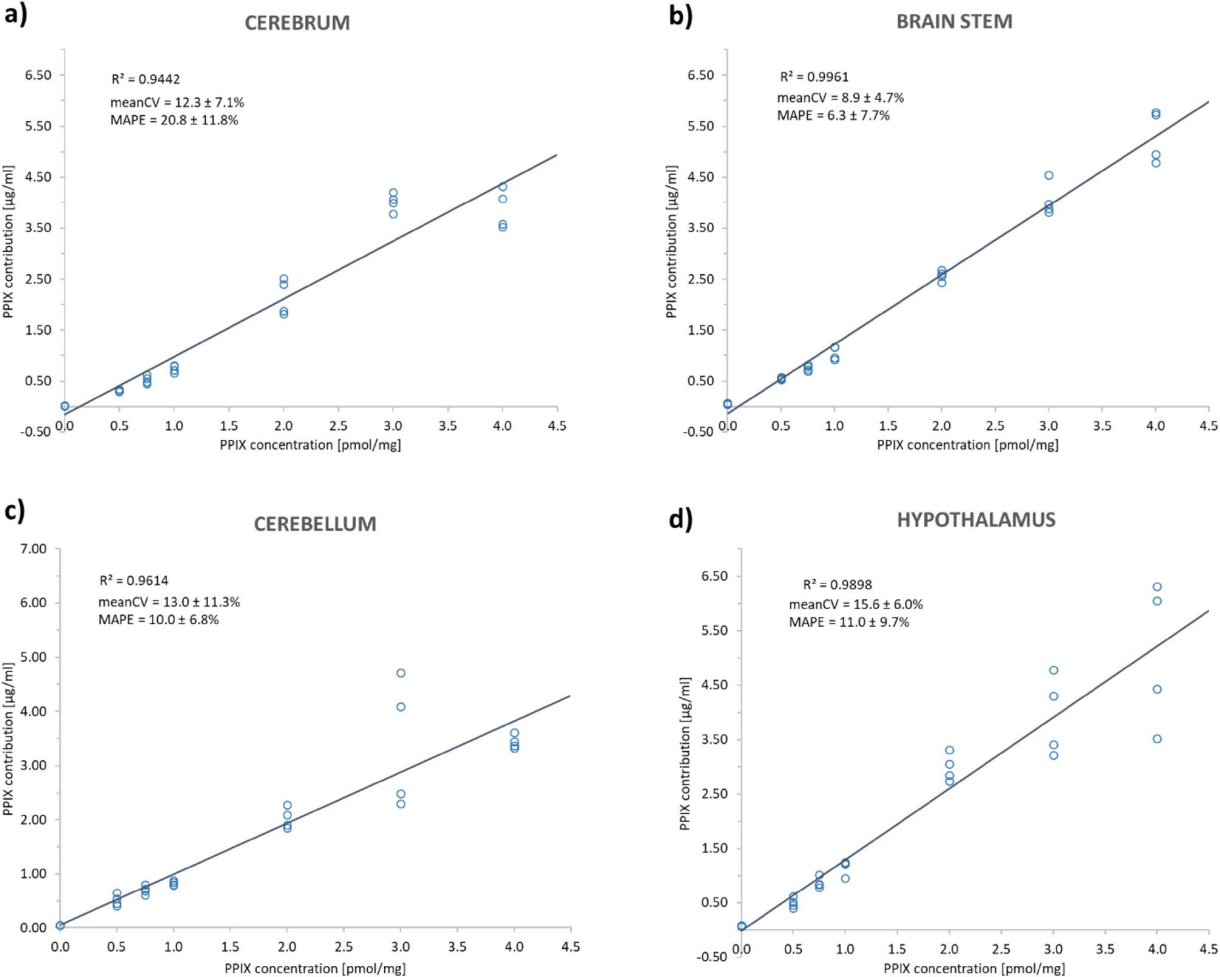
Results for the correlation of hyperspectrally measured PPIX contributions [µg/ml] with known PPIX concentrations [pmol/mg] in pig brain homogenates (CV = coefficient of variation, MAPE = mean absolute percentage error, mean ± standard deviation). Homogenates were prepared from (**a**) cerebrum, (**b**) brain stem, (**c**) cerebellum and (**d**) hypothalamus to accommodate variations of tissue optical properties. PPIX was spiked in known concentrations: 0.0, 0.5, 0.75, 1.0, 2.0, 3.0 and 4.0 pmol/mg with n = 4 replicates.

**Figure 4:**
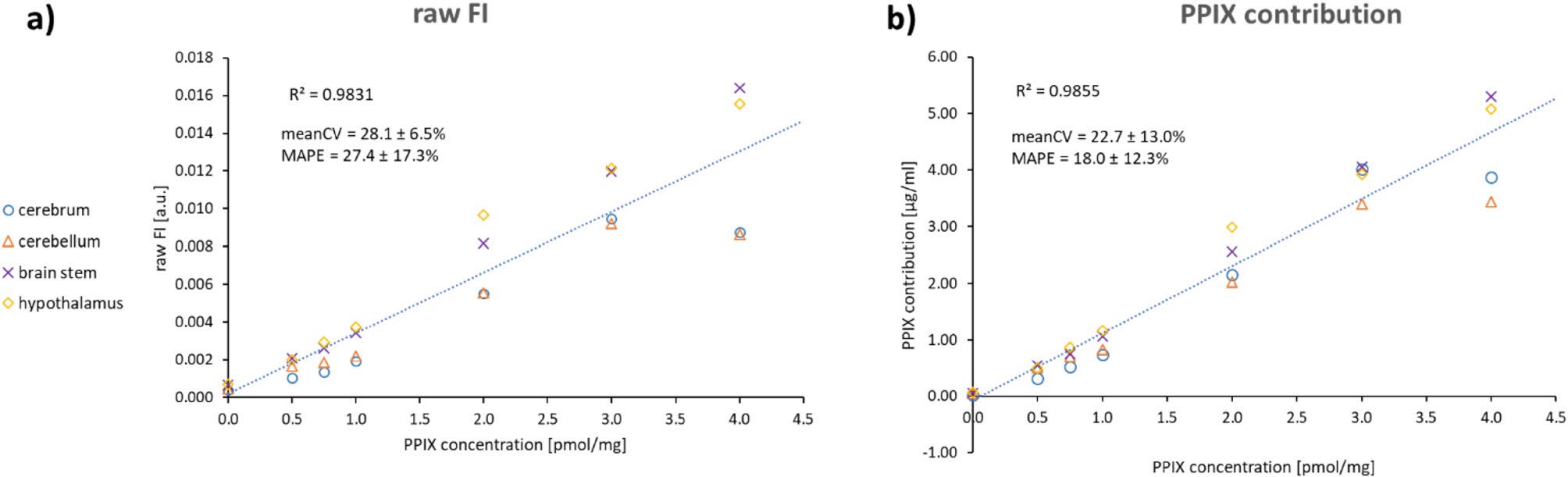
Results for the correlation of (**a**) raw fluorescence intensity (FI) and (**b**) calculated PPIX contribution [µg/ml] with known PPIX concentration [pmol/mg] (CV = coefficient of variation, MAPE = mean absolute percentage error, mean ± standard deviation) for all four investigated pig brain sections.

We used homogenates from pig brains to evaluate the HI system, including the algorithm developed by Valdes et al.^28,41^ for signal processing of hyperspectral data. This algorithm relies on liquid tissue-simulating phantoms for calibration. These phantoms are composed of intralipid as a scattering agent, yellow dye for absorption and PPIX^26,28,35^ and are thus only surrogates in mimicking tissue properties. Therefore, we tested the performance of HI based on PPIX-spiked brain homogenates prepared from cerebrum, cerebellum, brain stem and hypothalamus. The use of the raw fluorescence to determine PPIX was compared to the PPIX contribution as determined by complete signal processing to evaluate the impact of the algorithm. We found a linear correlation for each processing state of the data: (1) raw fluorescence, (2) corrected fluorescence after normalization, (3) relative fluorophore contribution and (4) calculated absolute contribution to the spiked PPIX concentration (for analysis steps, see supplementary Figure S1; for data, Figs. 3 and 4).

The mean absolute percentage error (MAPE) was calculated as a measure of prediction accuracy for the linear measurement curve obtained from correlation of the hyperspectrally measured PPIX contribution (Figs. 3, 4b) or the raw fluorescence intensity (FI, Fig. 4a) with the spiked PPIX concentration. MAPE was slightly lower when independently evaluating homogenates from each brain region (Fig. 3) compared to a model that used all four homogenates simultaneously (Fig. 4b). Comparing the values obtained from raw FI and PPIX contribution (Fig. 4), the use of the PPIX contribution was superior. Mean coefficient of variation (CV), MAPE, and coefficient of determination (R^2^) were improved using the processed and more specific PPIX contribution compared to the raw FI. The mean CV in the measured PPIX contribution for replicate (n = 4) preparations and measurements of homogenates varied from 8.9 ± 4.7% in brain stem to 15.6 ± 6.0% in hypothalamus. Additionally, during pig brain experiments, the hyperspectrally calculated PPIX contribution followed a linear trend above the upper limit of the calibration range in phantoms (2.5 µg/ml). Thus, extrapolation of the upper limit of the calibration for evaluation of pig brain homogenates and clinical biopsies was accepted.

**Figure 5:**
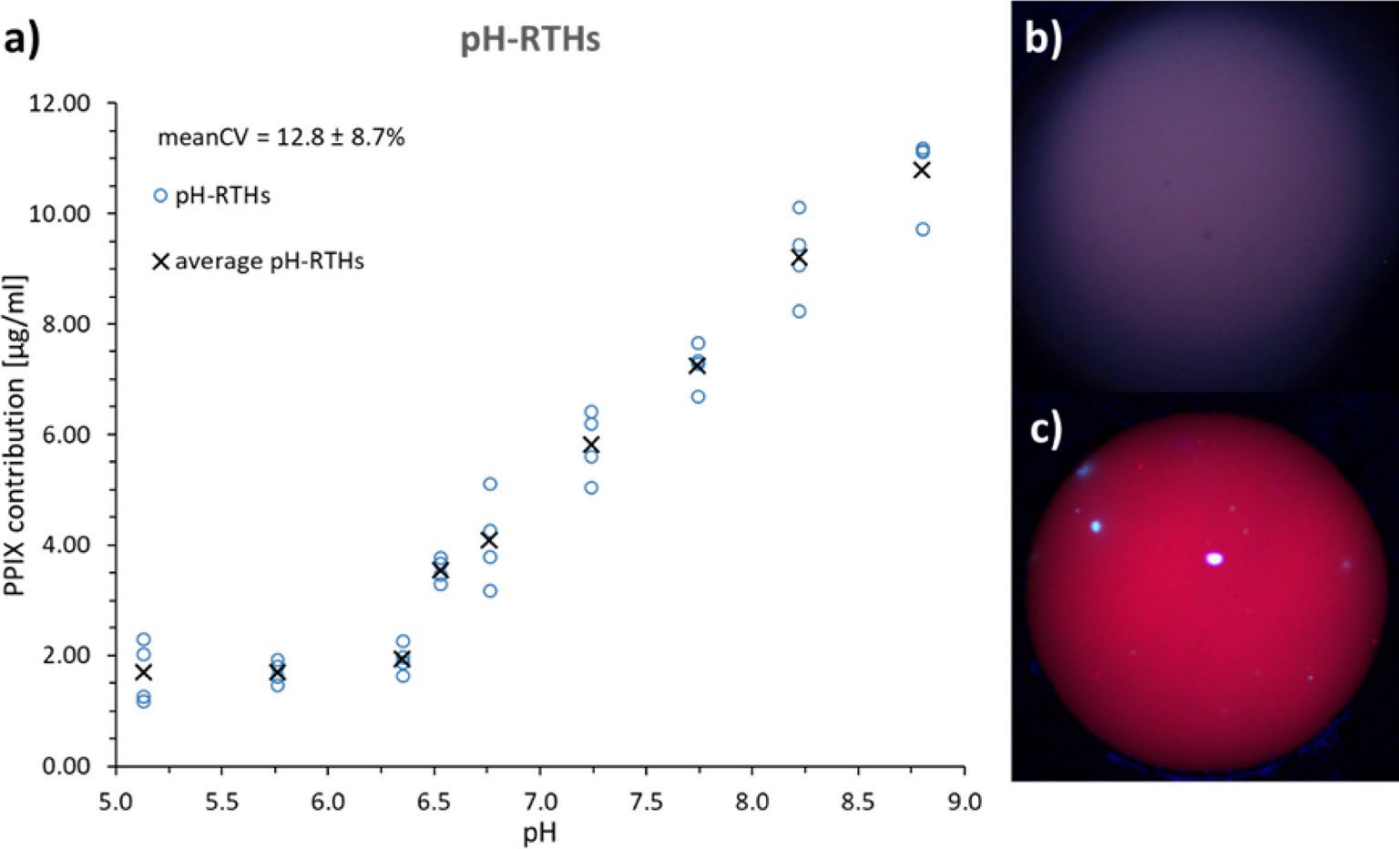
pH-dependence of the measured PPIX contribution as determined with pH-RTHs (PPIX concentration 3.0 pmol/mg). (**a**) PPIX contribution varied strongly with pH (meanCV = mean coefficient of variation ± standard deviation, n = 4). (**b**/**c**) BLUE 400 images for (**b**) pH 5.1, (**c**) pH 8.8.

We also studied the influence of pH in cerebrum homogenates because Alston et al.^31^ described a nearly fivefold increase in the fluorescence intensity for an aqueous solution of PPIX when shifting the pH from 5 to 9. We detected sixfold more PPIX at pH 8.8 (10.80 ± 0.71 µg/ml) than at pH 5.1 (1.70 ± 0.56 µg/ml) for pH-RTHs (Fig. 5a, supplementary Table S1). The visible fluorescence was also enhanced (Fig. 5b, c).

### Biopsy evaluation with respect to experience from former HI projects

**Figure 6:**
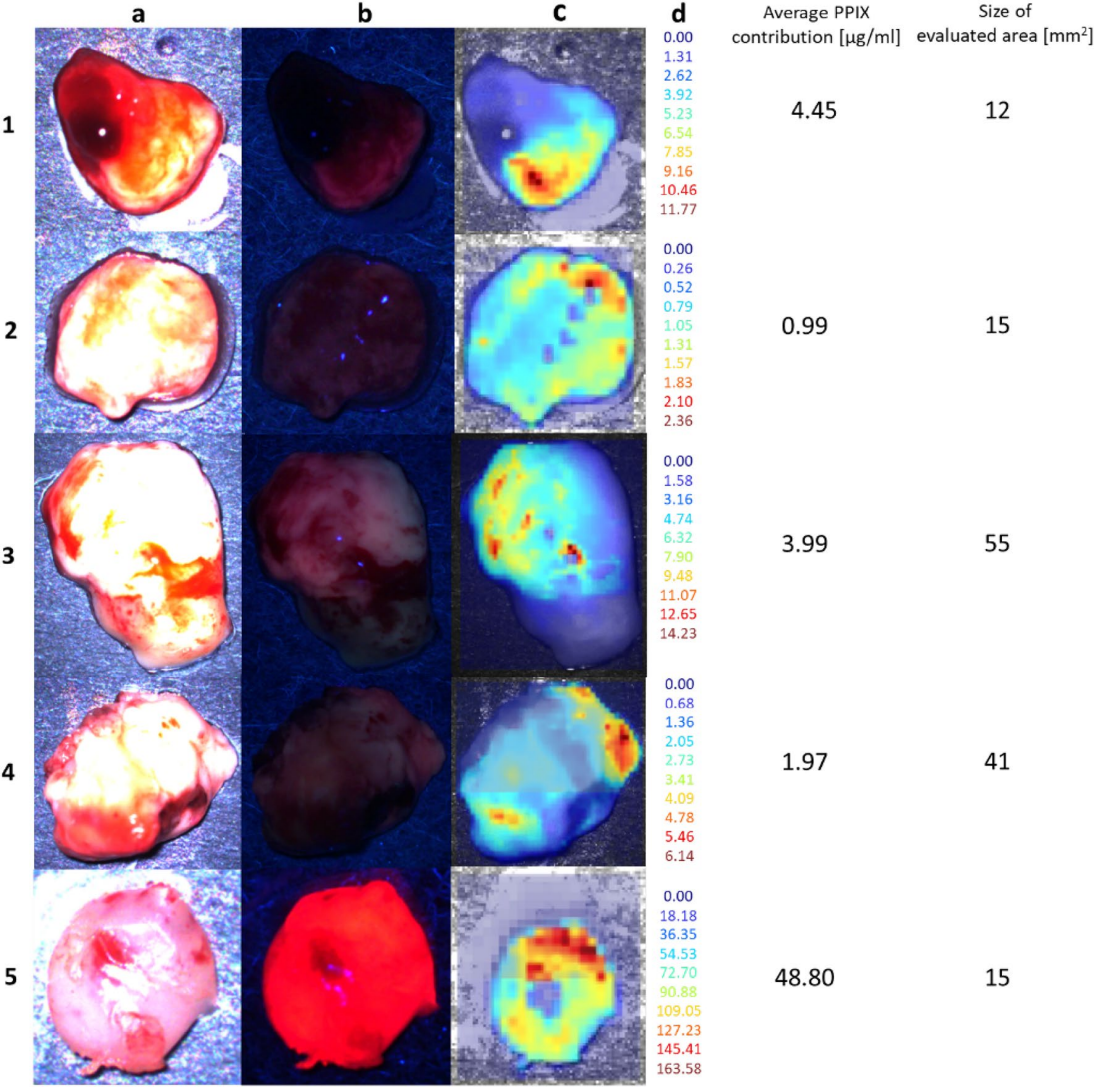
Hyperspectral images of biopsies. (**a**) Color image, (**b**) BLUE 400, (**c**) Hyperspectral overlay, (**d**) PPIX contribution [µg/ml] per pixel. Average PPIX contribution [µg/ml] and the evaluated tissue area size are given. (1) IZ biopsy with no visible fluorescence in the microscope; hyperspectral measurement visualizes PPIX fluorescence and residual blood. (2) ST biopsy of small size with pixels of high reflectance and underestimated PPIX contribution. Microscope fluorescence rating was “none”, whereas, in hyperspectral imaging, weak fluorescence was visible (2b). (3 and 4) RABT samples of middle size, fluorescence quality “weak” in the microscope. Both showed heterogeneous PPIX distribution. (5) Tiny ST biopsy showing strong fluorescence in the surgical microscope and an artificially high PPIX contribution in hyperspectral measurement of up to 164 µg/ml.

Initially, 245 samples were measured with HI, but five biopsies had to be excluded later. One biopsy (solid tumor (ST), fluorescence quality “weak”) could not be evaluated because of its tiny size and high specular reflectance. Three more biopsies also showed high specular reflectance, which led to overcompensation during normalization, giving low calculated PPIX contributions in these pixels. With most pixels suffering from this effect in these three biopsies, calculating a reliable average was impossible (see, e.g., Fig. 6, biopsy 5). In a fifth sample, residual blood was observed, covering PPIX fluorescence (Fig. 6, biopsy 1).

Determination of a representative average PPIX contribution (avPPIX) was challenging for biopsies with a very heterogeneous PPIX distribution and several individual PPIX hot spots (Fig. 6 biopsy 3 and 4). Thus, three strategies for evaluation were tested. First, the whole biopsy was selected for average calculation without a threshold using the software selection tool. This analysis yielded low avPPIX values, especially compared to biopsies with homogeneous but less intense PPIX fluorescence. Second, avPPIX was calculated as above, but using the highest native PPIX contribution obtained from pig brain homogenate experiments as a threshold (0.1 µg/ml) to exclude the native background. Third, regions of interest (ROI) were selected manually, including only the most intense PPIX spots in the average calculation. The third approach yielded the highest avPPIX values, whereas the second was a good compromise between the first and the third (for illustration see supplementary Figure S2).

### Device comparison

**Figure 7:**
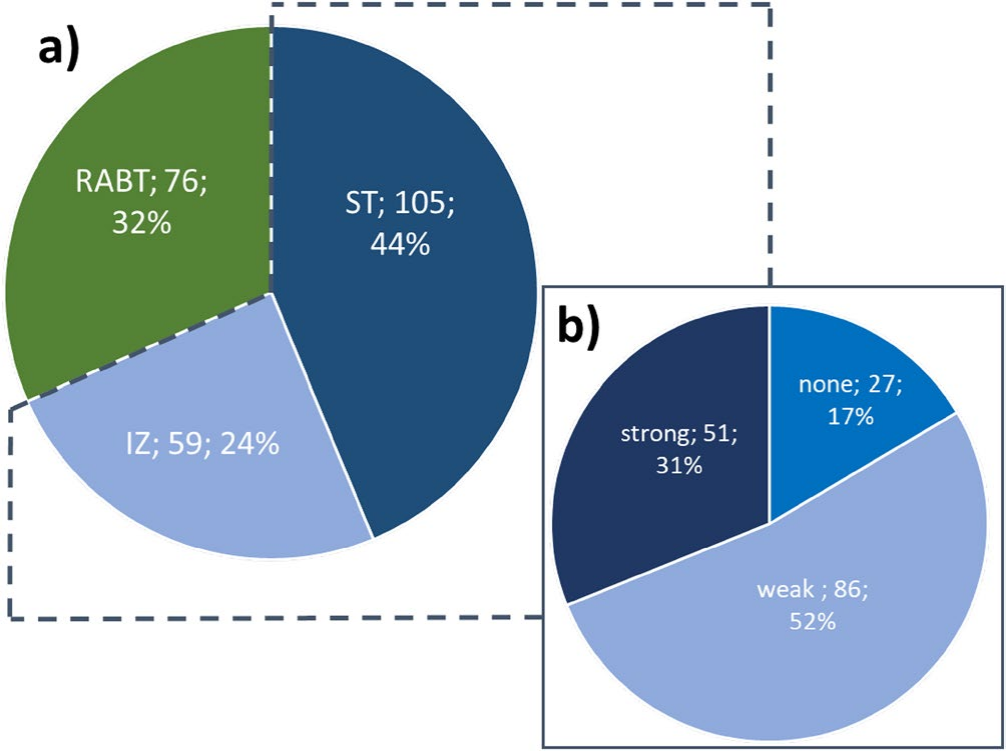
(**a**) Neuropathological assessment of the entire sample set, 240 biopsies. (**b**) Fluorescence qualities observed with the surgical microscope in ST and IZ biopsies (n = 164).

Two hundred forty biopsies were evaluated, and neuropathological and fluorescence results are depicted in Fig. 7. For 17% (n = 27) of histopathologically confirmed IZ and ST samples, no fluorescence was observed with the surgical microscope (Fig. 7).

**Figure 8:**
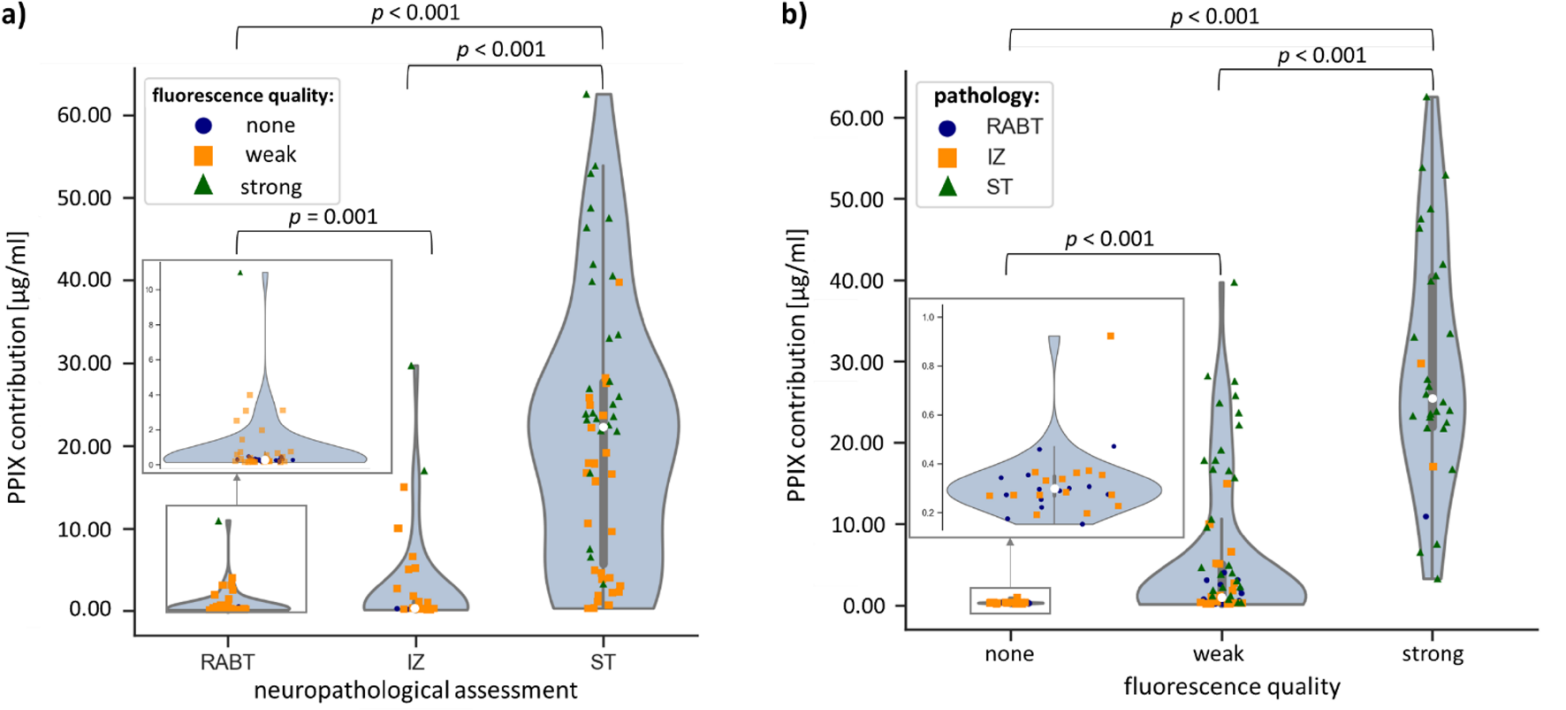
PPIX contributions in the cohort of 240 samples. (**a**) neuropathological grouping and (**b**) groups according to the quality of fluorescence as observed with the surgical microscope. Inside the violin graphs, bars show the interquartile range (IQR), whiskers represent 1.5-fold IQR, the white dot is the median of the distribution.

The avPPIX for the different biopsy groups (RABT, IZ, ST) is shown in Table 2 and Fig. 8. The distribution of the values varied between the groups (Kolmogorov–Smirnov *p* < 0.05). Groups differed significantly (MWU-test: RABT vs. IZ: U = 1515.50, *p* = 0.001; RABT vs. ST: U = 413.00, *p* < 0.001; IZ vs. ST: U = 879.00, *p* < 0.001). The avPPIX for ST presented with fivefold higher PPIX values than for IZ. Their ranges still overlapped; in all these groups, biopsies showed weak fluorescence. Minimum avPPIX was the same for all groups and equal to the threshold set for average calculation (0.1 µg/ml, Table 2). Nevertheless, pixels were present in these biopsies with lower PPIX contributions than 0.1 µg/ml. When considering the grouping according to fluorescence quality, strongly fluorescing biopsies had a considerably higher minimum PPIX value (2.2 µg/ml) than samples of the other two categories, where the minimal average value remained at 0.1 µg/ml.

**Table 2:** Average PPIX contribution for groups formed according to fluorescence quality in the surgical microscope and results of the neuropathological assessment (range, average ± standard deviation [µg/ml]).

Neuropathological assessment	Range of PPIX contribution [µg/ml]	AvPPIX ± standard deviation [µg/ml]	N
RABT	0.14–11.00	0.79 ± 1.55	76
IZ	0.15–29.69	3.06 ± 5.36	59
ST	0.16–62.56	16.33 ± 14.27	105
Fluorescence quality			
None	0.14–0.99	0.31 ± 0.18	62
Weak	0.14–39.71	6.25 ± 8.45	123
strong	2.16–62.56	21.24 ± 15.71	55

**Figure 9:**
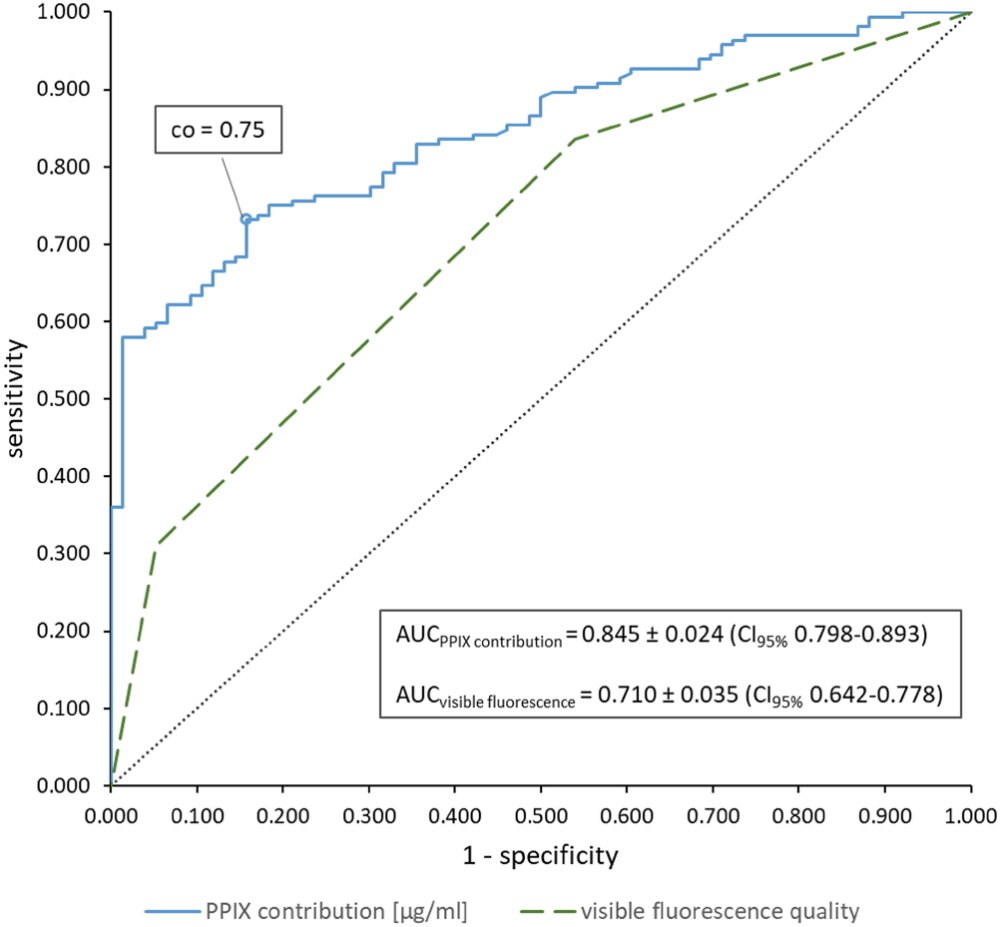
ROC analysis to find the optimal cut-off value (co) for the hyperspectrally measured PPIX contribution for diagnosis of tumor tissue (blue solid) and comparison with the visible observed fluorescence quality in the surgical microscope (green dashed) as diagnostic variables (black dotted = reference line).

Receiver operating characteristic (ROC) analysis was performed to find the optimal cut-off value for hyperspectrally measured PPIX for diagnosing a tumor biopsy (Fig. 9). The point closest to the upper left corner of the blue curve was the cut-off value of 0.75 µg/ml providing 90.6% positive predictive value (PPV), 56.3% negative predictive value (NPV), 73.2% sensitivity and 84.2% specificity. Additionally, the accuracy of the diagnostic variables (i) visible fluorescence quality in the surgical microscope and (ii) hyperspectrally calculated avPPIX were compared with ROC analysis. The avPPIX (AUC = 0.845 ± 0.024, CI_95%_ 0.798–0.893) was superior to the visible fluorescence estimation in the surgical microscope (AUC = 0.710 ± 0.035, CI_95%_ 0.6420.778) for diagnosing tumor biopsies (Fig. 9). Pairwise comparison of ROC curves according to the method by DeLong et al.^45^ showed a significant difference (AUC: 0.135 ± 0.0274, CI_95%_ 0.0817–0.189, z statistic 4.940, *p* < 0.0001).

In addition to ROC analysis, the threshold for qualitative visible fluorescence in the surgical microscope was determined using descriptive statistics in the biopsy group “none” (Table 2, Fig. 8b). The maximum avPPIX in this group was 0.99 µg/ml (Table 2), which is considered the threshold for visible fluorescence. As expected, conflicts between fluorescence evaluation from the surgical microscope and histopathology mainly occurred for weakly fluorescing biopsies (grades “none” or “weak”). Most weakly fluorescing biopsies showed avPPIX above the threshold for qualitative visible fluorescence (65/86, 76% in IZ/ST; 73/123, 59% for all samples).

Compared to the diagnostic cut-off value determined by ROC analysis (co = 0.75 µg/ml), the threshold for visible fluorescence (0.99 µg/ml) was higher. When this threshold was considered as cut-off for diagnosing tumorous tissue, PPV was slightly decreased, specificity was equal, but sensitivity and NPV were decreased (Table 3). Especially IZ biopsies were susceptible to a false-negative evaluation leading to an NPV of 59.3% (co = 0.75 µg/ml). When calculated only for RABT and ST biopsies, the NPV was 88.9% (co = 0.75 µg/ml).

**Table 3:** Variables for diagnostic performance of the hyperspectral camera as a function of different cut-off PPIX contributions.

Cut-off [µg/ml]	Sensitivity [%]	Specificity [%]	PPV [%]	NPV [%]
0.99	70.7	84.2	90.6	57.1
0.75	73.2	84.2	90.9	59.3

## Discussion

In this study, we compare the fluorescence evaluation with a surgical microscope to data obtained from a HI system for 240 biopsies from 26 patients undergoing FGR for HGG. Based on our extensive experience^18–24,30^, we thereby question established procedures such as using tissue-simulating liquid phantoms for calibration and the data analysis algorithm. Lessons have been learned from every process stage, including biopsy evaluation with the surgical microscope, validation of the hyperspectral method with pig brain homogenates, and assessment of heterogeneous biopsies.

### Ex vivo biopsy evaluation with the surgical microscope

The evaluation of the visible tissue fluorescence is highly subjective. Considerable training and intraoperative experience with PPIX fluorescence are critical to effectively and safely perform FGR^46,47^. External light sources and reflections impact the visible fluorescence impression; thus, they are dimmed as much as possible during surgery. Blood-covering tissue hampers fluorescence detection, even when significant amounts of PPIX are present (Fig. 6, biopsy 1). This effect is due to the fact that hemoglobin shares its core structure with PPIX and absorbs light around 405 nm^33,48–50^. It is thus essential to remove the blood from the tissue before fluorescence detection. Generally, as determined by HI, PPIX fluorescence in the surgical microscope is visible above a threshold of 0.99 µg/ml^23^.

### Dual-band normalization technique for HI signal processing

The detected tissue fluorescence is a function of the fluorescence emitted by the fluorophore of interest (e.g., PPIX) and other critical factors such as tissue optical properties and autofluorescence^16,36,37^. Methods that correct the total raw fluorescence either explicitly measure tissue optical properties using single-point spectroscopy or multispectral band imaging^26,51,52^, or use empiric measurements that can serve as surrogates for tissue optical properties^16,36^. One fluorescence correction method is an empirical spectrally constrained dual-band normalization technique published by Valdes et al.^28,41^. We have used this method with our HI system and recently refined the spectral unmixing by adding lipofuscin and flavin base spectra to correct for multiple autofluorescence sources^24^. Compared to unmixing with only the NADH base spectra, the fitting error in areas with PPIX contributions below 1 µg/ml was reduced by 82.4% for the spectral region of 420–730 nm and 92.3% less false-positive PPIX identifications were recorded in the control group^24^.

Areas with specular reflection or areas with an acute change in angulation provide unreliable underestimates of the PPIX contribution (e.g., Fig. 6, biopsies 1, 2 and 5) when using any fluorescence correction algorithm, including the dual-band normalization technique^36,41^. We thus note several limitations to our current methodology. First, our hyperspectral system for ex vivo imaging does not use polarized filters, which would be necessary to minimize specular reflection^53^. Second, our methodology needs to adequately account for the 3D profiles of tissue, which could be achieved using techniques such as stereo imaging^54^. Inadequate estimation of tissue profiles leads to inaccurate PPIX estimates and in these cases, using any fluorescence correction algorithm leads to inaccurate results^36,55^. So far, it has been essential to detect as much light as possible on the camera sensor and keep the system robust so that these issues need to be tackled in future devices.

The method developed by Valdes et al.^28,41^ for signal processing of hyperspectral data proved its robustness. Initially, dual-band normalization was created and validated in tissue-mimicking liquid phantoms with different optical properties. The root mean square error and the coefficient of determination for a linear calibration model were improved using the estimated PPIX contribution compared to the raw FI^41^. Our results in pig brain homogenates confirmed these observations. The coefficient of determination and MAPE were likewise improved by evaluating brain homogenates from different brain regions with the more specific PPIX contribution. To the best of our knowledge, the brain homogenates experiments are the first validation attempts for the dual-band normalization in brain tissue.

### Calibration for hyperspectral PPIX quantification

In experiment 1 (Fig. 1), we address the problem that current algorithms for evaluating HI data are based on device calibration with tissue-mimicking liquid phantoms. These phantoms have several drawbacks: they have a pH between 4–5, which is low compared to that of normal brain and glioma tissue (pH 6–8). Moreover, they do not provide different PPIX photo state distributions and contain only the fluorophore PPIX. According to analytical chemistry principles, one would choose a matrix similar to the real sample for device calibration.

The phantoms significantly impact on the “optical range of validity” of fluorescence correction algorithms, such as the dual-band normalization. The algorithm is only verified in the provided range of optical properties of the phantoms, e.g., absorption and scattering. It is, however, essential to cover the full range of optical properties expected in normal brain and tumor tissue. All measurements by an imaging system will depend on the accuracy of the assumptions and “ground truth” values (e.g., PPIX contribution) of these benchmarks^56–58^. The dual-band normalization technique used for fluorescence correction was explicitly developed for PPIX imaging using liquid phantoms. With these phantoms, i.e., the yellow dye, it is assumed that the absorption at the excitation wavelength is much larger than at the emission^28,41^. The selected range of absorption and scattering at both the excitation and emission wavelengths are approximated to those found in normal brain and brain tumors. Still, there is a discrepancy between the reduced scattering reported for normal brain and tumor tissue at the emission wavelength (µ’_s,635 nm_ = 9–100 cm^-1^)^59–63^ and the range displayed by phantoms (µ’_s,635 nm_ = 9–15 cm^-1^)^28,35,41^. However, the expected absorption range at the excitation wavelength is good but not perfectly covered (normal brain and tumor tissue^59–63^: µ_a,405 nm_ 5–50 cm^-1^; phantoms^28,35,41^: µ_a,405 nm_ 18–60 cm^-1^).

Phantoms provide only one PPIX-photo state at 634 nm (PPIX_634_) and have an acidic pH of about 4–5 without any pH variation. They do not accommodate for any known PPIX photo product or compounds causing tissue autofluorescence^35,41^. A second PPIX-photo state with a fluorescence maximum at 620 nm (PPIX_620_) was, however, described in neurosurgical applications^24,32^. The distribution of PPIX_620_/PPIX_634_ is believed to be dependent on the microenvironment, e.g., pH and macromolecule concentration^32,64,65^. The quantum yield of both PPIX photo states differs significantly. Thus, the photo state distribution is essential for quantification using the observed fluorescence and simple broad-band integration biases the PPIX concentration^32^.

The algorithm's performance concerning the correction of the matrix optical properties and pH was assessed by using pig brain homogenates. We did not yet replace the phantoms for instrument calibration during technical instrument modification because new calibration standards, e.g., tissue homogenates, need careful testing of their applicability and superiority compared with current standards. Measurements of PPIX contributions in single brain section homogenates were feasible with good reproducibility (mean CV 8.9 ± 4.7–15.6 ± 6.0%). The variation of optical properties by using different tissue matrices was sufficiently corrected using the method developed by Valdes et al.^28,41^ (Fig. 4). In contrast, pH significantly influenced the determined PPIX contribution, which could not be accounted for during signal processing^28,41^. The explored pH range of 5–9 in this and other work^43^ exceeded the physiological range of pH 6–8 assumed in normal brain and glioma tissue^66,67^. Still, even in this range, the PPIX contribution changed fourfold. Moreover, the differences between intra- and extracellular pH in tumor tissue^66,67^ may play a role in the in vivo photo state distribution dependent on the cellular localization of PPIX. Thus, there are limitations to PPIX quantification with the current method.

Some of the limitations of the phantoms can be overcome. For example, adaption of the range of maximum and minimum scattering and absorption values should be reconsidered to cover all possible ranges intrinsic to tissue without unrealistically increasing the range. An absorbing agent such as hemoglobin could better mimic the absorption observed in tissues. Further, a phantom with a more significant number of relevant fluorophores would extend the accuracy limits of the technique. Attempts in using the dual-band normalization accounted for PPIX, its photo products, and in part for autofluorescence with a linearly decreasing fit at wavelengths > 600 nm^35,41^. Recent studies have shown that using both photo states and multiple individual autofluorescing compounds during spectral decomposition improves already the accuracy of tumor detection^24,32^. In aqueous phantoms, the pH and the amounts of lipid and surfactant impact on the PPIX-photo state distribution^64,65,68^. To that end, it is essential to cover the full range of possible optical properties and variations in biochemical microenvironment, e.g., pH^31^. Calibration samples composed of brain homogenates, pH-buffer and the known compounds to adjust optical properties, e.g., intralipid and yellow dye, may serve this purpose and combine the advantages from phantoms, especially the precise adjustment of an extended range of optical properties, with those from brain homogenates.

### Strategies for improved description of heterogeneous biopsies

Hyperspectral wide-field imaging allows the analysis of the PPIX contribution in each pixel within the field of view. Thus, cameras with low- to sub-millimeter resolution display a detailed map of PPIX contribution for the imaged tissue^14,16,18,28,38,69^.

Fluorescence trials to validate the hyperspectral technique rely on evaluating measured PPIX contributions, neuropathology assessments, magnetic resonance imaging and observed fluorescence quality in the surgical microscope. A single avPPIX value is calculated to describe the whole biopsy^17,20,25,32,34,39,70^. In highly heterogeneous biopsies with multiple hot zones, as shown in Fig. 6, biopsies 3 and 4, a single avPPIX may not represent the whole sample adequately. Therefore, we tested different approaches for evaluation and finally introduced a threshold into the average calculation to exclude pixels with PPIX contributions lower than 0.1 µg/ml. This was the maximum native PPIX contribution measured with our hyperspectral device for pig brain homogenates. This threshold accounts for spatially separated PPIX hot zones and then the avPPIX represented a suitable compromise (supplementary Figure S2). Thus, to avoid bias due to subjective user-dependent decisions, we recommend the selection of the entire biopsy with the software selection tool for avPPIX determination, along with using a proper threshold for discrimination of the non-fluorescing background.

Ultimately, quantitative hyperspectral fluorescence imaging in glioma surgery aims to create an accurate and reproducible map of PPIX contributions across the surgical field of view in vivo to predict infiltrative regions and assist the surgeon in performing a complete tumor resection. Determining the best method for calculating representative avPPIX in ex vivo biopsies is of more academic than practical use from the perspective of surgical workflow. Still, it is essential for fluorescence trials that will ultimately rely on these data.

### Histopathological assessment

In an earlier study^29^, several false-positive biopsies (RABT with weak or even strong fluorescence in the surgical microscope and partly above the threshold for visible fluorescence of 0.99 µg/ml) were observed. Initially, staining was only performed with H&E, p53, Ki67/MIB-Index and, only when applicable, IDH-1. Predominantly with the help of IDH staining, the samples were re-assessed. Since most of these tumors were IDH-1 mutated, 8 of 15 biopsies could be diagnosed as IZ due to visualization of additional tumor cells. Therefore, re-evaluation of the cohort samples in our study was carried out. However, the few patients with false-positive samples harbored IDH wild-type glioblastomas, so further IDH staining was not helpful. Out of 45 re-evaluated biopsies, four were re-assigned as IZ by preparation of serial cuts. In three false-positive IDH mutant biopsies, additional tumor cells could be depicted by IDH staining, but not enough to change the diagnosis to IZ. This raises the question of histopathological reference standards.

### Predictors for diagnostic accuracy and threshold for visual detection of fluorescence in the surgical microscope

In this study, the PPV of the hyperspectral device was 90.9% (co = 0.75 µg/ml); similar studies^25–27,39^ found an average PPV of 87% (range: 70–100%) for the hyperspectral technique.

The NPV depends strongly on the sampling strategy for non-fluorescing biopsies^17,46^. Here, the NPV for the hyperspectral device was 59.3% (co = 0.75 µg/ml). Especially IZ biopsies were susceptible to a false-negative evaluation. Consequently, the NPV was higher (88.9%, co = 0.75 µg/ml) when calculated only for RABT and ST biopsies. Others^25–27,39^ found an average NPV of 68% (range: 50–87%). Each false-negative biopsy had PPIX contributions below the threshold for visible fluorescence in the surgical microscope of 0.99 µg/ml. As noted in the introduction, the lower PPIX levels can be multifactorial, including, but not limited to, low tumor cell density^17^ and low proliferation rates^70^. Another important reason for the possible discrepancy between PPIX levels and neuropathological assessment could be that tumor cells producing PPIX might be buried within the tissue. Thus, the given excitation light at 405 nm with a penetration depth of about 100—250 µm only reaches the tissue surface. The surgical microscope and the hyperspectral system cannot excite PPIX much below the surface^69,71,72^.

The results from the present sample cohort were consistent with previous data from our group, which suggested a threshold for visible fluorescence with the surgical microscope of 1 µg/ml^22,23^, and with work by Valdes et al.^26,27,70^ coming to the same conclusion. However, it is essential to note that visible fluorescence can still be observed below 1 µg/ml, given the effects of tissue optical properties and pH. Here, 28% of the fluorescing biopsies had PPIX contributions below 1 µg/ml.

Comparison of the hyperspectral technique with conventional surgical microscopy showed that the hyperspectral camera was superior for diagnosing tumorous tissue (AUC = 0.845 ± 0.024, CI_95%_ 0.798—0.893 vs. AUC = 0.710 ± 0.035, CI_95%_ 0.642—0.778) due to the lower sensitivity and less specific PPIX fluorescence detection with the surgical microscope. The threshold for visible fluorescence depiction in the surgical microscope was 0.99 µg/ml, and thus higher than the optimal cut-off value for diagnosing tumorous tissue by HI (0.75 µg/ml). HI is, therefore, a promising tool for glioma surgery and should be further improved for clinical application.

## Conclusion

HI can diagnose tumors more sensitively than surgical microscopy in malignant glioma biopsies obtained during FGR with 5-ALA. However, the technique still needs to be improved for surgical application, which is challenging predominantly due to the need for real-time PPIX data processing. Intraoperative real-time imaging brings new challenges compared to biopsy imaging in a laboratory setting and requires an adapted HI setup. Here, we have scrutinized several aspects of PPIX determination in biopsies. We present improvements in experiment and data analysis, which contribute to increase sensitivity and specificity. So far, correction in the HI analysis algorithm for variations in tissue optical properties is sufficient, but further effort is required to accommodate variations in biochemical microenvironment, multiple autofluorescence sources and native absorbing agents, e.g., blood, into samples intended for calibration and validation. For this purpose, combining the established phantoms with brain homogenates for device calibration seems promising. Further effort is required to transfer hyperspectral devices from research to clinic.

## Supplementary Information

Supplementary Information 1.

Supplementary Information 2.
