# Supplementary material for: Challenges in, and recommendations for, hyperspectral imaging in ex vivo malignant glioma biopsy measurements
Source: Sci Rep. 2023 Mar 7;13:3829. doi: 10.1038/s41598-023-30680-2 (PMC9992662; doi:10.1038/s41598-023-30680-2)
Supplement: Supplementary file 1 — Supplementary Information 1. [file 41598_2023_30680_MOESM1_ESM.docx]

**Challenges in, and recommendations for, hyperspectral imaging in *ex vivo* malignant glioma biopsy measurements – Supplementary information**

Anna Walke^1,2^, David Black^3^, Pablo A Valdes^4^, Walter Stummer^1^, Simone König^2^, Eric Suero-Molina^1^

*^1^Department of Neurosurgery, University Hospital of Münster, Münster, Germany.*

*^2^Core Unit Proteomics, Interdisciplinary Centre for Clinical Research, University of Münster, Münster, Germany.*

*^3^Department of Electrical and Computer Engineering, University of British Columbia, Vancouver, Canada.*

*^4^Department of Neurosurgery, University of Texas Medical Branch, Galveston, TX, United States.*


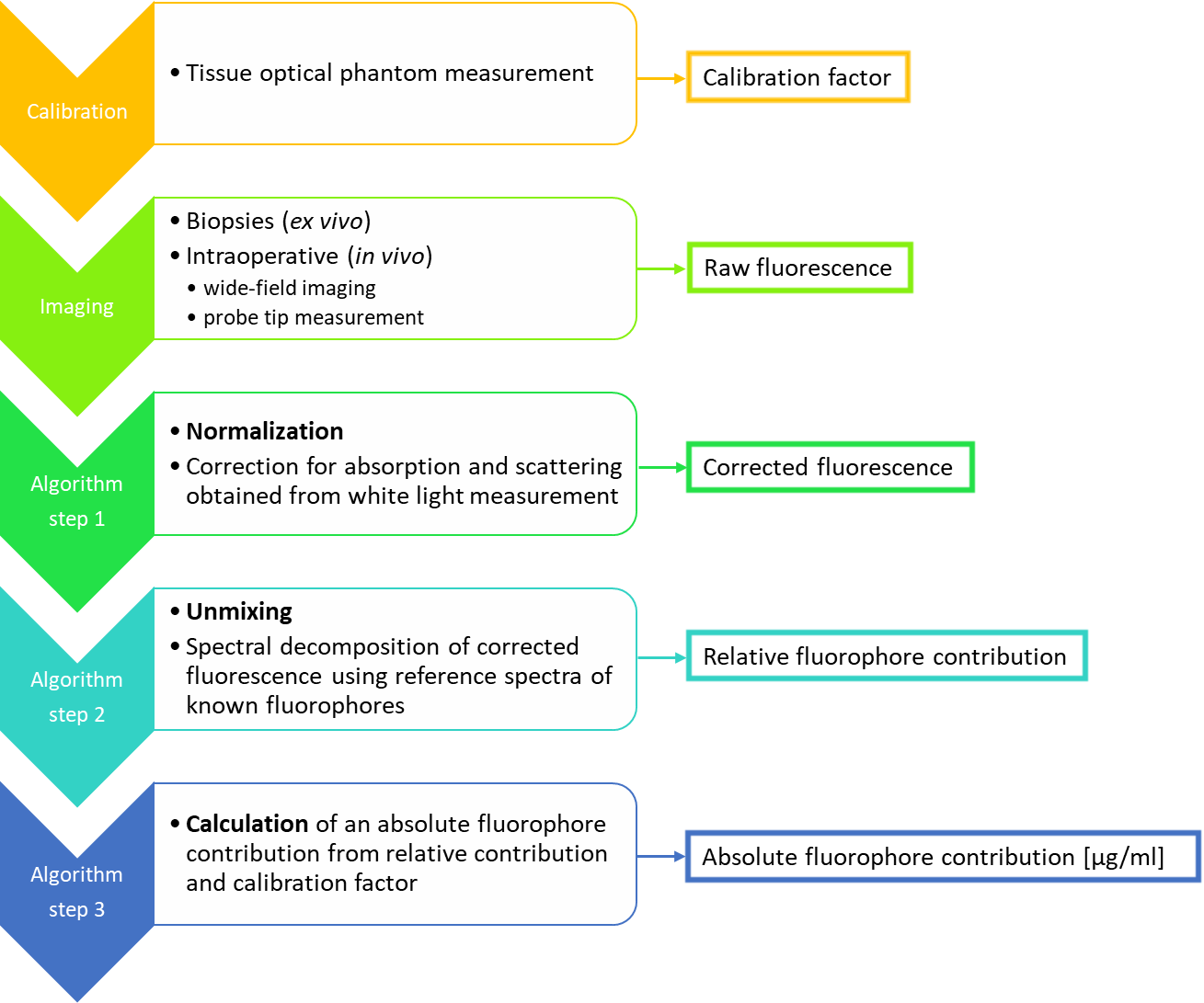


**Figure S1:** Workflow for a hyperspectral experiment according to Valdes et al.^[[1]](#footnote-1)^ First, the device needs to be calibrated, a process that spectroscopy specialists currently perform right after technical modification of the system rather than in daily research application. Routinely, tissue-mimicking phantoms are used for this purpose. Intraoperative imaging or biopsy imaging generates fluorescence values, which need to be corrected for distorting tissue effects, e.g., absorption and scattering (normalization, algorithm step 1). Spectral unmixing (algorithm step 2) subsequently isolates the PPIX signal, which is then used to calculate the analyte contribution (algorithm step 3).

**Table S1:** Composition of pH-RTHs. In * pH was adjusted again with HCl after mixing Tris buffer with RTH.

| **Tris buffer pH after adjustment with HCl** | **Final Tris concentration [M]** | **RTH [mg]** | **Addition of Tris buffer [µl]** | **Final pH** | **PPIX concentration [pmol/mg]** |
| --- | --- | --- | --- | --- | --- |
| **5.0** | 0.5 | 600 | 600 | 5.1* | 3.0 |
| **5.5** |  |  |  | 5.8* |  |
| **6.0** |  |  |  | 6.4* |  |
| **6.5** |  |  |  | 6.5 |  |
| **7.0** |  |  |  | 6.8 |  |
| **7.5** |  |  |  | 7.2 |  |
| **8.0** |  |  |  | 7.7 |  |
| **8.5** |  |  |  | 8.2 |  |
| **9.0** |  |  |  | 8.8 |  |


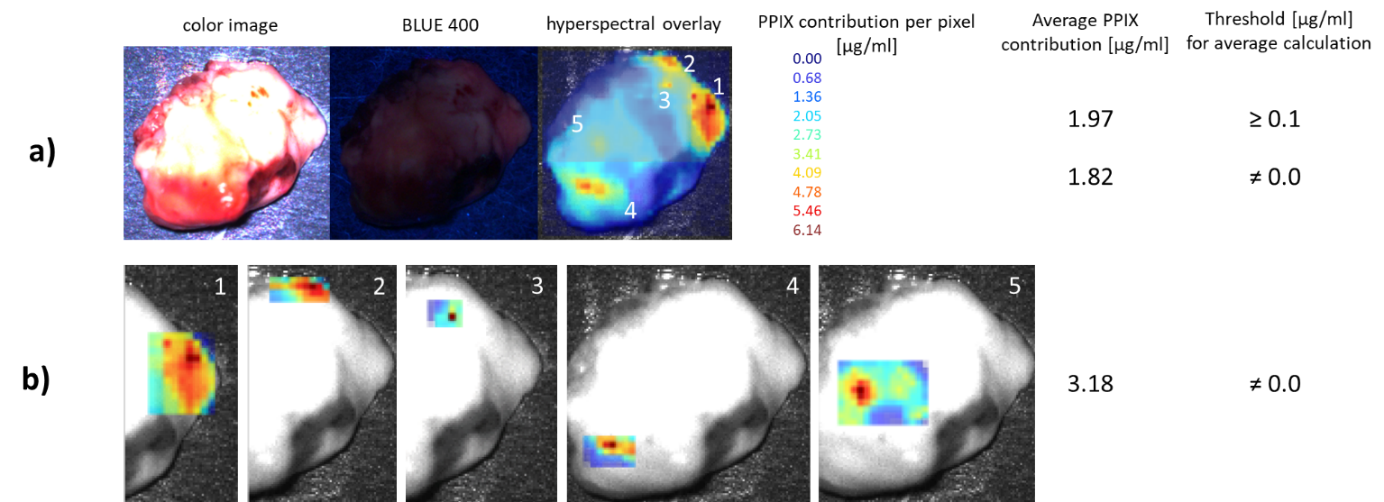


**Figure S2:** Analysis of a heterogeneous sample with several distinct PPIX hot spots (manuscript Figure 6, biopsy 4). a) ROI - entire biopsy, using different thresholds to exclude pixels from the calculation. b) Hyperspectral overlays showing manually selected ROIs for avPPIX calculation.

1. Valdes, P.A. *et al*. Quantitative, spectrally-resolved intraoperative fluorescence imaging. *Sci. Rep.* (2012);

   Valdes, P.A. *et al.* A spectrally constrained dual-band normalization technique for protoporphyrin IX quantification in fluorescence-guided surgery. *Opt. Lett.* (2012); see manuscript for full reference citation. [↑](#footnote-ref-1)
